# Thyroid Extract for Myxœdema. II

**Published:** 1892-07-23

**Authors:** 


### The Practitioner's Mirror and Retrospect.

[*The Editor will be glad to receive offers of co-operation and contributions from members of the profession. All letters should be addressed to* The Editor, The Lodge, Porchester Square, London, W.]

### MEDICINE.

### THYROID EXTRACT FOR MYXŒDEMA.—II.

Dr. George Murray, at the annual meeting of the British Medical Association in 1891 reported the results he had obtained from the injection of fresh thyroid juice in a myxœdematous patient under his care. He remarks that if we consider that myxœdema and the cachexia strumipriva are due to the absence from the body of some substance which is present in the normal thyroid gland, and which is necessary to maintain the body in health, it is at least rational treatment to supply that deficiency as far as possible by injecting the extract of a healthy gland. His method is as follows: The lobe of the thyroid gland of a sheep is removed as soon as possible after the animal has been killed. The surrounding fat and connective tissue are removed from it. All the instruments and glass vessels used in the further preparation of the extract should be either sterilised by heat or thoroughly cleansed with a one in twenty solution of carbolic acid. The gland is cut up on a glass dish into small pieces, and then placed in a test tube with one cubic centimetre of pure glycerine, and one cubic centimetre of a 0·5 per cent. solution of carbolic acid. The mouth of a tube is closed with a plug of cotton wool and the mixture allowed to stand in a cool place for twenty-four hours. The mixture is then placed in a fine handkerchief which has previously been placed for a few minutes in boiling water. It is then firmly squeezed by screwing up the handkerchief so as to express as much liquid as possible from the handkerchief. By this means Dr. Murray found that three cubic centimetres of a turbid pink liquid are obtained. This preparation will keep quite fresh for at least a week.

The patient was a female aged 46. Four or five years previous to her coming under the treatment her friends had noticed that her speech and actions were becoming very slow. She herself began to feel soon after that it required a great effort to do her ordinary housework. Her features gradually became enlarged and thickened. The hands and feet increased in size and became altered in shape, and for four years she had not perspired at all, and only once menstruated. She presented the characteristic features of myxœdema.

Three months later, when she had had the extracts of five lobes of sheep's thyroid injected, that altogether representing the extract of two and a half glands, the patient had steadily improved. Though three weeks were allowed to elapse between the injection of the last two extracts, she did not lose any of the ground she had previously gained. The swelling had gradually diminished, and had practically disappeared from the backs of the hands, the skin over them being now loose and freely movable. The lips were much smaller, and the swelling of the upper eyelids so much diminished that she could look up quite easily, instead of scarcely at all as on admission. The speech became more rapid and fluent. She answered questions more readily, the memory improved, and she found that it required much less effort than formerly to do her work in the house.

In his concluding remarks Dr. Murray adds: "Many cases of myxædema doubtless do improve to a certain extent when untreated, and it is not wise to draw many conclusions from a single case; but the return of perspiration and menstruation, when they have not occurred for four years, together with the many other signs of improvement which have followed the treatment, are, I think, sufficient indication that this treatment really has a beneficial effect upon the disease. The improvement, of course, cannot be expected to be continued if the injections are discontinued, but there seems no reason why it should not be maintained if the injections are repeated at intervals of two or three weeks.

A more recent case treated by these injections was that of a lady, aged 45, under Dr. Beatty, of Dublin, who reports that on examining the case it was quite clear that she was suffering from myxædema. Her face was swollen, waxy looking, and anæmic; there was a well-marked and circumscribed pink flush on her cheeks, contrasting markedly with the pale swollen skin of the rest of the face. The eyelids were swollen and looked œdematous. The alæ nasi were thickened and the nose broadened. The lips were thickened, and the tongue seemed too large for her mouth, and evidently interfered somewhat with articulation. The hands were enlarged and clumsy. The extract of thyroid glands were prepared in the manner indicated by Dr. Murray, and extract from the two lobes of one thyroid gland was given in three parts, with two days' interval between. The patient experienced no unpleasant sensations. The extracts of five thyroid glands were given. Each extract was given in three separate injections within a week after its preparation. The time between the administration of each set of injections has varied from four to ten days. The effect of the injections was truly marvellous, says Dr. Beatty. A marked improvement in the patient's condition was noticeable within one week. The improvement steadily progressed, so that in six months Dr. Beatty was able to state that the patient was practically cured; the face looked natural, the skin of the face no longer thickened, the eyelids not swollen, her movements active, hair growing naturally, memory returned. "No physician seeing her now could recognise her as one of myxædema," wrote Dr. Beatty.

Dr. Carter* reports a case of a woman, aged 43, who was insane, and who was also suffering from well marked myxœdema. She was injected with twenty-five minims of the extract of the thyroid gland of a cow, prepared according to the directions of Dr. Murray. Most of the subsequent injections were made from the pig's thyroid. They were repeated twice a week for nearly four months. The mental symptoms improved, and rapid progress took place in improvement of the myxœdematous symptoms.

The time cannot be far distant when the relative value of transplantation and injection will be urged by their respective upholders. Certainly, these few reported cases allow us to hope that the treatment of myxœdema is no longer hopeless, although definite statements in our present position would be premature. We have accordingly, as far as possible, confined our remarks to simple facts.

* *British Medical Journal*, April 16th, 1892.

